# Effect of Milk Thistle (*Silybum marianum*) Supplementation on Pork Offal Quality

**DOI:** 10.3390/ani12121526

**Published:** 2022-06-13

**Authors:** Kinga Kropiwiec-Domańska, Marek Babicz, Monika Kędzierska-Matysek, Magdalena Szyndler-Nędza, Ewa Skrzypczak, Bartłomiej Woliński

**Affiliations:** 1 Department of Animal Breeding and Agriculture Advisory, University of Life Sciences in Lublin, Akademicka 13, 20-950 Lublin, Poland; marek.babicz@up.lublin.pl (M.B.); barwolt97@gmail.com (B.W.); 2 Department of Quality Assessment and Processing of Animal Products, University of Life Sciences in Lublin, Akademicka 13, 20-950 Lublin, Poland; monika.matysek@up.lublin.pl; 3 Department of Animal Genetics and Breeding, National Research Institute of Animal Production, ul. Krakowska 1, 32-083 Balice, Poland; magdalena.szyndler@izoo.krakow.pl; 4 Department of Animal Breeding and Product Quality Assessment, Poznań University of Life Sciences, Słoneczna 1, 62-002 Suchy Las, Poland; ewa.skrzypczak@up.poznan.pl

**Keywords:** fattener pig, offal, pork quality, feed, milk thistle

## Abstract

**Simple Summary:**

Due to their natural origin and a wide range of positive effects on the body, herbs and spices are currently becoming commonly used as a nonantibiotic component of pig rations. There is research evidence to confirm health-promoting effects of milk thistle on humans and animals. The greatest effects are seen for the liver, i.e., protection from toxins and regeneration. The aim of the study was to determine the effect of adding milk thistle seeds on the quality of edible internal organs from the fatteners, which are used in food production as offal. We suggest that our study shows room for improvement of offal quality and may have a positive impact on consumer awareness in the context of offal consumption.

**Abstract:**

The aim of the study was to determine the effect of milk thistle supplementation of fattener pig feeds on physical and chemical properties of pork offal. The experiments were conducted on 60 fatteners (group C—control (30 pigs) and group E—experimental (30 pigs)). The experimental group was supplemented with ground milk thistle (*Silybum marianum*) at 7 g/kg feed. The offal (tongues, kidneys, hearts, lungs and livers) was analyzed for weight, pH, WHC, water, protein, fat, energy value, fatty acid profile and content of major and trace elements. The present study shows that milk thistle added to fattener pig diets increased pH45 and pH24 values in most of the analyzed offal and significantly (*p* ≤ 0.01) decreased the weight of heart and lungs and increased the weight of liver and kidneys. Hearts, lungs and kidneys of the experimental group contained more fat and the liver less, than the same offal of the control group. As regards the content of elements, the dietary supplement most often had an effect on the heart and lungs. In general, milk thistle supplemented in fattener diets had modified the physical parameters and chemical composition of the analyzed products.

## 1. Introduction

The European Union ban on in-feed antibiotics as growth stimulants in 2006 has created a trend for supplementing pig feeds with biologically active substances, which may have beneficial effects on their health and production results while increasing the nutritional value and taste qualities of pork [1,2]. Therefore, phytobiotics, or organic chemical compounds from herbs, spices, fruit and vegetables are increasingly used as feed supplements [3,4].

The positive effects of herbs and spices derive from the high content of biologically active substances such as alkaloids, glycosides, flavonoids, essential oils, terpenes, organic acids and vitamins [5]. They boost the immune system and show anti-stress, antibacterial, antiviral, anti-inflammatory and antifungal activities [6]. One such herb is milk thistle (*Silybum marianum* [L.] *Gaertn*). Due to its specific chemical composition and hepatoprotective effect, milk thistle is increasingly used to feed farm animals raised under intensive conditions [7,8]. The health-giving nature of this plant arises from the high content of biologically active compounds, e.g., taxifolin, quercetin, kaempferol, apigenin and eriodyctiol. The main (1.5–3% dry matter), biologically active component of milk thistle seed coat is silymarin. It is an active complex of flavonolignans composed of silibinin (60%), isosilibinin (5%), silidianin (20%) and silicristin (10%) [9]. The silymarin complex shows a number of positive functions for the body: it scavenges free radicals, inhibits the formation of prostaglandins and inflammatory leukotrienes, regulates glutathione absorption and cell membrane permeability, which protects the liver from toxins entering hepatocytes [10,11]. Furthermore, it promotes rRNA synthesis and inhibits the transformation of hepatic stellate cells into myofibroblasts, thus regenerating the liver and protecting it from cirrhosis and fibrosis [12]. Silymarin reduces the expression of cytokine TGF-beta, normalizes blood sugar levels in hyperglycemic patients, aids in the treatment of stress-induced stomach ulcers, and shows hypocholesterolemic and antiatherogenic effects [13,14]. It also has antihemorrhagic and antithrombotic properties, strengthens blood vessel walls and stimulates the secretion of bile and stomach juices [9]. It also prevents the formation of gallstones and removes heavy metals from the body [15,16]. 

The quality of pork raw material: meat, fat and offal depend on genetic and environmental factors. The most important of these are diet and breed [17,18,19]. 

The most popular pork offal are kidneys, lungs, heart, liver and tongue. In the food industry they are most often used to produce offal products (or ready-made stuffing. Some offal may be consumed directly after preparation and heat treatment [20]. In this context it is important to monitor the nutritive value, which is crucial for human dietetics and nutrition and reflected not only in the proportion of the main chemical components but also in the lipid profile and the content of major and trace elements [21]. 

In view of the increasing use of phytobiotics in pig nutrition, it is interesting to understand the technological and eating quality of offal obtained from fatteners.

The aim of the study was to determine the effect of milk thistle supplementation of fatteners feed on physical parameters and chemical composition of pork offal.

## 2. Materials and Methods

### 2.1. Animals

The experiment was conducted with a group of 60 Pulawska pigs at an initial body weight of 25 ± 1.5 kg. Animals were kept in compliance with animal welfare requirements [22]. The study did not require the approval of the Ethics Committee and conformed with Directive 2010/63/EU on the protection of animals used for scientific purposes [23]. There was no legal basis for requesting approval from the Local Ethics Committee to conduct the research because in accordance with Article 2, Paragraph 4, Point f of Directive 2010/63/EU [23] and Article 2, Paragraph 1, Point 6 of Journal of Laws 2015 item 266 [24] and during the research no activities were performed that could cause pain, suffering, stress or permanent damage to the body equal to or more than a needle stick injury. Experimental procedures were not performed on live animals. Offal for evaluation was taken from animals after slaughter. The animals were slaughtered in accordance with routine procedures of the slaughterhouse. 

Furthermore, according to the law in force in Europe and Poland [25,26] milk thistle is a feed additive approved for use in feeding pigs. Therefore, the feed used satisfied all the nutritional needs of the animals and did not cause any clinical anomaly or pain, so according to Annex no 1 [23] the applied nutrition was not a procedure requiring the approval of the Commission.

Another argument in favor of the lack of the need for approval of the Ethics Committee for the research is that the purpose of our study was not to determine the permissible maximum dose of milk thistle in pig nutrition but to determine the effect of a non-toxic amount of milk thistle supplement in pig nutrition on the consumer quality of offal (see Appendix A). Barrows were divided into two groups: C—control (30 pigs) and E—experimental (30 pigs), All animals had *ad libitum* access to feed mixture (Table 1) and water. Single-phase feeding to appetite in a dry system was used. The experimental group (E) was supplemented with ground milk thistle (*Silybum marianum*) at 7 g/kg feed. 

The milk thistle dose resulted from a previous preliminary experiment with 30 fatteners at an initial body weight of 25 kg. Animals (3 groups with 10 pigs per group) were fed until the end of fattening (around 110 kg) with a diet supplemented with 5 g (group 5), 7 g (group 7) or 10 g (group 10) milk thistle per kg feed. Animals were weighed twice weekly to calculate daily gains. Daily feed intake was also determined. The fatteners fed the diet supplemented with 7 g of milk thistle were characterized by intermediate feed intake and higher daily gain (Table 2). 

The ground milk thistle used in the experiment contained 16.5% protein, 19.6% fat, 24.2% crude fiber, 2.93% silymarin complex, and the following active substances: silybin 1.82%, isosilybin 0.37%, silydianin 0.01%, silycrystin 0.73%. The amount of major and trace elements was as follows: 5370 mg K, 360 mg Na, 600 mg Ca, 313 mg Mg, 20,400 mg Mn, 75,100 mg Fe, 54 µg Pb, 129 µg Cd. Milk thistle composition was determined according to standard AOAC procedures [27]. Silymarin content was determined using a PerkinElmer HPLC system with a 235C DAD detector on a Hypersil BDS C 18 column (PerkinElmer LAS GmbH, Rodgau, Germany). 

### 2.2. Slaughter

The slaughtering was carried out in September. All pigs were slaughtered after 114 days of the experiment. Mean slaughter weight, fattening results and slaughter value are presented in Table 3.

Pigs were transported to the meat processing plant located 98 km away, in accordance with the provisions of Council regulation (EC) [28]. After a 3-h rest period animals were automatically electrically stunned (250 V, 5 A, 2.4 s) and slaughtered in accordance with the regulations of the meat plant. On the date of sale, fatteners were subjected to a standard health evaluation by the veterinary surgeon supervising the herd. After slaughter, in keeping with the meat plant procedure [29] carcasses with offal were subjected to veterinary assessment to determine suitability of the meat and offal for consumption.

### 2.3. Preparation of Samples for Laboratory Tests

Samples of whole organs (liver (60 pcs), lungs (60 pcs), tongue (60 pcs), kidneys (60 pcs) and heart (60 pcs)) were collected for laboratory analysis. The choice of the samples was determined by the consumer acceptance of certain domestic pig organs as edible offal [30,31]. This prepared offal was weighed and then and transported in portable refrigerators (+4 °C) to the laboratory.

#### Determination of Physical Chemical Properties

Determination of the physical properties of whole offal included pH and water holding capacity (WHC). pH was measured 45 min and 24 h after slaughter using a pH-Star CPU device (Matthäus, Germany) and WHC [32,33].

The samples for chemical tests were homogenized in a Büchi Mixer B-400 (Flawil, Switzerland). The percentage of basic chemical components (water, protein, fat) was measured with a Foss FoodScanTM Meat analyzer using near-infrared (NIR) spectroscopy in accordance with standard PN-A-82109 [34]. The calculations of energy value (kJ·100 g^−1^ of fresh tissue) were based on Atwater equivalents, where 1 protein = 4.0 kcal = 16.76 kJ; 1 g fat = 9.0 kcal = 37.66 kJ.

Total fat for analysis of the fatty acid profile was extracted with a chloroform/methanol mixture according to the method of Folch et al. [35]. Further fatty acid profile tests were conducted according to standards PN-EN ISO 5508 [36] and PN-EN ISO 5509 [37]. Fatty acids were analyzed as methyl esters using a Varian 450-GC gas chromatograph (Varian Inc., Walnut Creek, CA, USA) with a flame ionization detector (FID) fitted with CP-8400 Autosampler. Particular fatty acids were expressed as a percentage of all fatty acids identified. Taking account of the degree of fatty acids saturation, they were grouped as saturated fatty acids (SFA), unsaturated fatty acids (UFA), monounsaturated fatty acids (MUFA), polyunsaturated fatty acids (PUFA), omega-3 polyunsaturated fatty acids (OMEGA-3), and omega-6 polyunsaturated fatty acids (OMEGA-6). In addition, calculations were made for the UFA/SFA, MUFA/SFA PUFA/SFA ratios as well as of the content of neutral and hypocholesterolemic fatty acids (DFA = UFA + C18:0) and hypercholesterolemic fatty acids (OFA = SFA − C18:0). 

The lipid quality indices of atherogenicity (AI) and thrombogenicity (TI) were calculated following the equations of Ulbricht and Southgate [38].

The content of major elements: potassium (K), sodium (Na), magnesium (Mg), calcium (Ca) and trace elements: zinc (Zn), iron (Fe), manganese (Mn), copper (Cu), were determined by flame atomic absorption spectrometry (FAAS; air-acetylene flame) using a Varian AA240FS Fast Sequential Atomic Absorption Spectrometer (Varian Australia Pty Ltd., Sydney, Australia). A 1 g portion of each sample was placed in CEM MARSXpress (PFA) vessels, 5 mL of 65% nitric acid (Suprapur) (Merck, Darmstadt, Germany) was added and then digested for 30 min using a MarsXpress microwave oven (CEM Corporation, Mattews, NC, USA). After digestion, the digestate was transferred to 25 mL volumetric flasks and supplemented with deionized water. 

To eliminate interferences, the samples for determination of K, Na, Ca and Mg were diluted with Schinkel correction buffer (10 g L^−1^ CsCl + 100 g L^−1^ La).

The accuracy of the determinations were verified by measuring blank samples and Standard Reference Material 1577c BovineLiver (NIST, Gaithersburg, MD, USA). The concentration of elements in the samples were expressed in mg·kg^−1^ of wet weight.

### 2.4. Statistical Analysis

Analysis of the physical and chemical characteristics of the studied offal was performed. The normality of distribution was determined using the Kolmogorov-Smirnov test, and the homogeneity of variance was verified with Levene’s test. Tukey’s test was used for one-way comparison of means at *p* ≤ 0.01 and *p* ≤ 0.05. Statistical analysis was performed with SAS ver. 9.4 (SAS Institute Inc., Cary, NC, USA) using model (GLM) procedure for analysis of variance:

yij = µ + ki + eij

where:

yij—phenotypic value of a trait;
µ—population mean;
ki—fixed effect of feeding pigs (i = 1, 2);
eij—random error.

## 3. Results and Discussion

The performed analyses showed a statistically significant (*p* ≤ 0.01) effect of supplementing pig diets with milk thistle seeds on the weight of heart, lungs, liver and kidneys and on their percentage body weight of the pigs (Table 4). Heart and lungs from the experimental group weighed less than from the control group. Increased weight was also noted for the liver and kidneys. A significant relationship between feeding herbs to pigs and the weight of their internal organs is confirmed by other studies. Pietrzak and Grela [3] reported that dietary addition of 3.0% protein and xanthophyll concentrate from lucerne caused a significant increase (by around 10%) in liver and kidney weight. A considerable increase in liver weight (by around 34%) as a result of dietary supplementation of inulin and water/garlic extract was observed in (Polish Landrace × Polish Large White) × Duroc pigs weighing 110 ± 1.5 kg [39]. 

The values of basic physical parameters (pH45, pH24, WHC) are presented in Table 5. With regard to pH measured 45 min postmortem, significantly higher values were noted in offal from the experimental group in lungs (*p* ≤ 0.01), liver (*p* ≤ 0.05) and kidneys (*p* ≤ 0.01). For pH measured 24 h postmortem, higher values were also observed in the experimental group in tongue (*p* ≤ 0.01), lungs (*p* ≤ 0.01), liver (*p* ≤ 0.05) and kidneys (*p* ≤ 0.01). Other studies demonstrated that supplementation of animal diets with different forms and concentrations of milk thistle caused muscle pH to increase not only in pigs [7], but also in broiler chickens [40] and in rabbits [41]. This relationship may result from the hyperglycemic action of the active component of milk thistle, i.e., silibinin [42,43]. As reported by Colturato et al. [44], silibinin concentrations of 50–300 µM have a marked effect on carbohydrate metabolism in rat liver by inhibiting gluconeogenesis in the fasted condition as well as glycogenolysis and glycolysis in the fed condition. 

The supplementation of milk thistle seeds in pig diets caused a significant (*p* ≤ 0.01) decrease in WHC in the liver and an increase in kidneys. Physical properties combined with chemical composition of pork meat and offal determine not only the technological suitability but also the consumer attractiveness [45]. Table 5 shows the chemical composition of the offal analyzed in the experiment. Our study revealed a significant (*p* ≤ 0.01) effect of supplemental milk thistle on water content of the lungs (group C > group E). As regards the proportion of protein, a significant (*p* ≤ 0.01) effect of the supplementation was only noted for lungs (group C < group E) and kidneys (group C > group E). The highest protein content in both the control and experimental groups was characteristic of the liver, which is supported by other authors [21,46]. Fat is an important component of offal from the aspect of technological and eating quality. Our study results demonstrate that the dietary modification had a significant (*p* ≤ 0.01) effect on fat content in all the offal under analysis. Fatteners from the experimental group, compared to those from the control group, were characterized by significantly higher fat content in the tongue, heart, lungs and kidneys, and lower content in the liver. The decrease in liver fat content as a result of using milk thistle is consistent with the findings of Abenavoli et al. [47], who showed that in the diet of humans with nonalcoholic fatty liver disease, the milk thistle complex improved the biochemical profile of the liver and had a beneficial effect on the fat and glucose metabolism, thus reducing the liver fat content. 

The amount of protein and fat contributes to the energy value of a product (Table 5). Our statistical analysis showed a significant increase in the energy value of the lungs (*p* ≤ 0.01) and heart (*p* ≤ 0.05) in the experimental compared to the control group. 

Technological and nutritive value of a food product is determined not only by the amount, but also by the composition of fat, which is reflected in the fatty acids profile and in the percentage and ratios of their main groups [48]. 

The milk thistle supplement was shown to cause a statistically significant increase in the content of C16:0 and C14:0 acids (in the heart (*p* ≤ 0.05) and kidneys (*p* ≤ 0.01) as well as a decrease in the lungs (*p* ≤ 0.01) and liver (*p* ≤ 0.01) (Table 6). A significant (*p* ≤ 0.01) effect of the supplementation in all the analyzed offal was noted for C18:0 acid. Compared to group C, tongue, heart and lungs contained less, and liver and kidneys more stearic acid (Table 6). Stearic acid is named as a neutral and hypocholesterolemic acid [49] because it is easily transformed to unsaturated oleic acid (C18:1) in the human body, and thus does not increase blood cholesterol.

Among the polyunsaturated fatty acids in the offal, the highest concentrations were those of linoleic acid (LA, C18:2n-6) and α-linolenic acid (ALA, C18:3n-3) (Table 4). The share of these acids is particularly important because they are not synthesized by mammals. After absorption from food, these precursor acids are deposited in the muscle tissue or used in the liver and brain for synthesis of long-chain PUFA (LC PUFA), i.e., arachidonic acid (AA, C20:4n-6) and eicosapentanoic and docosahexaenoic acids (EPA, C20:5 n-3 and DHA, C22:6n-3 [50]. These derivatives play an essential role in human neurodevelopment because they are found primarily in cell membranes of the central nervous system (the brain and spinal cord) and take part in the synthesis of tissue hormones such as thromboxanes, leukotrienes, prostaglandins and prostacyclins [51].

Supplementation of the pig diets with ground milk thistle fruits caused a significant (*p* ≤ 0.01) increase in the LA content of the tongue and liver as well as a decrease in the kidneys. ALA was found to increase significantly (*p* ≤ 0.01) in all the analyzed offals, except for the lungs.

When analyzing the effect of supplemental milk thistle on the profile and proportion of fatty acids in the main groups, it was observed that supplementation of this phytobiotic caused a significant decrease in the content of saturated fatty acids (SFA) and increased unsaturated fatty acids (UFA) only in the tongue (*p* ≤ 0.05) and lungs (*p* ≤ 0.01) (Table 7). This supplement had no significant effect on SFA and UFA content in the heart, liver and kidneys. It is well to bear in mind, however, that the high proportion of unsaturated fatty acids in raw materials from pigs causes an unfavorable increase in oxidative susceptibility, including a shorter shelf life [52]. 

Analysis of the content of monounsaturated fatty acids (MUFA) showed that they increased significantly in the experimental group, notably in the lungs (*p* ≤ 0.01) and kidneys (*p* ≤ 0.01), followed by the tongue (*p* ≤ 0.05) and heart (*p* ≤ 0.05). Differences in MUFA content between the groups are due to a significant increase in the sum of cis and trans isomers of oleic acid (C18:1n9c + C18:1n9t, Table 6) in these offals. Out of all monounsaturated fatty acids, these isomers are present in offal at the highest concentrations, which is confirmed by other authors [20,21]. 

Another group of fatty acids are polyunsaturated fatty acids (PUFA). As reported by Davoli et al. [53], the meat industry prefers meats with low contents of PUFA because these negatively affect fat firmness and product quality, whereas consumers require higher contents of those fatty acids for their positive effect on human health. Statistical analysis showed a significant effect of the milk thistle supplement on increasing total PUFA, in particular OMEGA 6 in the tongue (*p* ≤ 0.01) and liver (*p* ≤ 0.05) and on decreasing these acids in the heart, lungs and kidneys (*p* ≤ 0.01) (Table 7). Frankiewicz et al. [54] observed a considerable increase in PUFA content in the meat and fat of pigs after dietary supplementation of the pigs with milk thistle seeds.

The dietary inclusion of milk thistle to pigs caused a significant (*p* ≤ 0.01) beneficial increase in DFA content and a decrease in OFA content in the lungs and liver. The content of DFA and OFA in the tongues, hearts and liver, observed in our study, is comparable to the values reported by Cebulska [55] for pork (DFA 73.70%; OFA 25.36%). 

From the perspective of human health, an important benchmark for fat quality is the PUFA to SFA ratio, which should be 0.4 or higher [56]. Our study showed that the offals with a favorable ratio of these acids were the liver and kidneys of the control group (Table 7). The milk thistle supplement caused a significant increase in PUFA/SFA ratio in the tongue (*p* ≤ 0.05) and a decrease in the heart (*p* ≤ 0.05) and kidneys (*p* ≤ 0.01).

Considering the medical aspects and the varying effects of different fatty acids on the human organism, Ulbricht and Southgate [38] distinguished two indicators that describe the atherogenic (AI) and thrombogenic (TI) potential of a diet more accurately than the ratio of different groups of acids. Tarricone et al. [57] report that lower AI and TI values are more beneficial for human health. In our study we observed that milk thistle supplemented to pig diets significantly (*p* ≤ 0.01) reduced TI value in the tongue, heart, lungs and kidneys. AI value was found to significantly decrease in the liver and lungs and to increase in the kidneys (Table 7). 

From the perspective of health and proper functioning of the human body, the amount of major and minor elements supplied by the diet is important. Sodium (Na), potassium (K), magnesium (Mg) and calcium (Ca) are the major elements necessary for normal development of the body, and their dietary requirement exceeds 100 mg per day [58]. Sodium regulates water/salt metabolism and aids in the transport of amino acids and carbohydrates into tissues. Together with potassium, it establishes a gradient across the cell plasma membrane, which allows for transfer of nerve impulses as well as contraction and relaxation of muscle fibers [59]. 

Potassium is antagonistic to calcium, which increases muscular tension and cell membrane permeability [50]. Another macro element is magnesium, which acts as a catalyst for protein metabolism as well as having effects on nerve conduction, the cardiovascular system and muscle contractility [60].

Trace elements in the human diet include iron (Fe), copper (Cu), zinc (Zn) and manganese (Mn). Iron is necessary for the normal function of the hematopoietic system [61]. Liver proved the best source of iron out of all offal under analysis. Our study showed that a 100-g piece of liver provides around 20 mg of iron, which is fifteen times as much as pork, around eight times as much as long-maturing hams [62], and six times as much as beef liver [31]. By analogy with iron, liver is the best source of zinc, copper and manganese, which is supported by other studies [21,46,63]. This is due to the biochemical function of liver and the fact that these trace elements are co-factors or components of many enzymes [64]. 

The comparative analysis results for the mineral content of offal, depending on diet are presented in Table 8. The inclusion of milk thistle into pig diets significantly (*p* ≤ 0.05) increased Na in the tongue and decreased it in the lungs of the pigs. The Na content in the tongue from our study is consistent with the results of Tomović et al. [46]. The Na content in the lungs is higher than the data reported by other authors [21,46,63].

For K it was observed that pigs from the experimental group had a significantly (*p* ≤ 0.01) lower concentration of this element in the heart and lungs as well as higher (*p* ≤ 0.05) concentration in the kidneys. 

The results obtained for the content of K in the heart of the control group are similar to those reported by the National Nutrient Database for Standard Reference Release [63] and Tomović et al. [46]. In the kidneys they observed lower K content than in our study, i.e., 2477 mg·kg^−1^ [46] and 2290 mg·kg^−1^ [63].

The addition of ground milk thistle fruits significantly (*p* ≤ 0.01) increased Ca content in the heart, lungs and liver. Tomović et al. [46], who studied autochthonous Swallow-Belly Mangalitsa pigs from Hungary noted mean Ca content to be lower in the heart (85.5 mg·kg^−1^), in the lungs (208.4 mg·kg^−1^) and liver (135.9 mg·kg^−1^) compared to our study.

The effect of milk thistle on increasing Mg concentration was noted in the liver (*p* ≤ 0.01). 

The Mg amounts noted in the control group were analogous to the results reported by other authors [21,46,63]. 

Our study (Table 8) showed that the milk thistle fruit supplement significantly increased the amount of Fe in the tongue, heart, lungs and liver (*p* ≤ 0.01). When comparing our findings to those of Tomović et al. (2016) we observed a similarity to the iron content in the tongue and liver of the experimental group, and to the heart and lungs of the control group. Supplementing fatteners with ground milk thistle seeds caused a significant decrease (*p* ≤ 0.01), in relation to the control group, in the Zn, Cu and Mn content of the heart, a decrease in the Zn content of the lungs, and an increase in Mn content of the lungs. The results obtained in our study for Zn content in the heart and lungs, in both the control and experimental groups, are slightly lower than the data reported by The US Department of Agriculture [63]. Cu content in the heart and Mn content in the lungs are similar to the findings of Tomović et al. [46], i.e., 3.2 mg·kg^−1^. 

## 4. Conclusions

The present study demonstrated that ground milk thistle seeds added to fattener diets significantly (*p* ≤ 0.01) decreased the weight of heart and lungs and increased the weight of liver and kidneys. In terms of physical characteristics, most of the offal from the experimental group showed significantly higher pH45 and pH24. Addition of ground milk thistle seeds to pigs’ diet caused a significant increase in the amount of fat in the kidneys, lungs, heart, and tongue, and reduced the amount of fat in the liver. Importantly, supplemental milk thistle seeds had a beneficial effect on the fatty acid profile, as evidenced by lower TI and AI values in the analyzed offal. As regards the content of major and minor elements, the dietary supplement most often had an effect on the heart and lungs.

In general, milk thistle supplemented in fattener diets modified the physical parameters and chemical composition of the analyzed products.

## Figures and Tables

**Table 1:** Composition and nutritional value of the feed mixture.

Specification	Composition
Ingrediens:	
Maize, %	18.00
Barley, %	28.00
Wheat, %	20.00
Soybean meal, %	16.00
Wheat bran, %	6.20
Rye bran, %	7.50
Premix, %	2.50
Rapeseed oil, %	1.25
Ground limestone %	0.30
Acidifier, %	0.25
Content in 1 kg of feed:	
Protein, %	15.00
Fat, %	2.2
Fibre, %	5.5
Methionine, %	0.26
Lysine, %	0.87
Metabolizable energy, MJ	12.7

**Table 2:** Results of the preliminary experiments.

Traits	Group		
	5 Mean ± SD	7 Mean ± SD	10 Mean ± SD
Daily feed intake, kg	3.01 ± 0.21	2.87 ± 0.17	2.75 ± 0.19
Daily gains during fattening, g/day	695.78 ± 8.30	777.12 ± 9.65	710.56 ± 7.91

**Table 3:** Fattening and slaughter value parameters.

Traits	Group	
	C Mean ± SD	E Mean ± SD
Mean slaughter weight, kg	113.92 ± 2.31	114.98 ± 3.19
Age of slaughter, days	178 ± 6.03	174 ± 7.82
Daily gains during fattening, g/day	780 ± 75	789 ± 67
Backfat thickness, mm	18.76 ± 2.97	19.01 ± 2.02
Loin eye height, mm	57.78 ± 4.33	58.28 ± 3.19
Meat content, %	55.17 ± 1.14	55.65 ± 1.76
Dressing percentage, %	78.75 ± 1.56	78.81 ± 1.77

C—control group; E—experimental group.

**Table 4:** Weight and share in pig body weight of the pigs.

Traits	Offal Type									
	Tongue		Heart		Lungs		Liver		Kidneys	
Groups	C mean ± SD	E mean ± SD	C mean ± SD	E mean ± SD	C mean ± SD	E mean ± SD	C mean ± SD	E mean ± SD	C mean ± SD	E mean ± SD
Weight, g	283 ± 24	279 ± 21	401 ^B^ ± 32	357 ^A^ ± 23	794 ^B^ ± 80	592 ^A^ ± 95	1417 ^A^ ± 85	1549 ^B^ ± 99	341 ^A^ ± 24	369 ^B^ ± 18
Share in pig body weight, %	0.25 ± 0.01	0.25 ± 0.01	0.36 ^B^ ± 0.02	0.32 ^A^ ± 0.02	0.71 ^B^ ± 0.11	0.53 ^A^ ± 0.08	1.27 ^A^ ± 0.09	1.38 ^B^ ± 0.12	0.30 ^A^ ± 0.02	0.33 ^B^ ± 0.01

C—control group; E—experimental group; A,B—significant differences in the columns for offal, marked with different capital letters, differ at *p* ≤ 0.01.

**Table 5:** Physical parameters, chemical composition and caloric content of the offal.

Traits	Offal Type									
	Tongue		Heart		Lungs		Liver		Kidneys	
	C Mean ± SD	E Mean ± SD	C Mean ± SD	E Mean ± SD	C Mean ± SD	E Mean ±SD	C Mean ± SD	E Mean ± SD	C Mean ± SD	E Mean ± SD
pH_45_	6.28 ± 0.34	6.30 ± 0.52	6.11 ± 0.17	6.13 ± 0.16	6.61 ^A^ ± 0.32	7.13 ^B^ ± 0.57	6.09 ^a^ ± 0.34	6.21 ^b^ ± 0.45	6.05 ^A^ ± 0.21	6.22 ^B^ ± 0.11
pH_24_	5.77 ^A^ ± 0.21	6.11 ^B^ ± 0.65	5.98 ± 0.31	6.04 ± 0.52	6.94 ^A^ ± 0.39	7.32 ^B^ ± 0.60	6.24 ^a^ ± 0.31	6.30 ^b^ ± 0.51	6.34 ^A^ ± 0.19	6.41 ^B^ ± 0.19
WHC, %	15.23 ± 1.95	14.99 ± 2.13	14.57 ± 2.88	15.31 ± 4.72	15.34 ± 1.65	15.78 ± 2.72	18.42 ^B^ ± 2.45	13.55 ^A^ ± 4.58	12.04 ^A^ ± 1.77	14.04 ^B^ ± 3.06
Water, %	66.97 ± 2.55	65.21 ± 4.95	74.88 ± 3.11	72.97 ± 6.15	77.08 ^B^ ± 4.84	73.87 ^A^ ± 6.69	70.69 ± 3.41	69.04 ± 3.76	76.04 ± 4.44	77.83 ± 3.04
Protein, %	16.67 ± 0.96	16.05 ± 1.62	16.39 ± 1.22	16.82 ± 1.22	18.02 ^A^ ± 1.83	20.93 ^B^ ± 2.77	22.64 ± 1.99	23.85 ± 1.87	17.63 ^B^ ± 2.12	15.78 ^A^ ± 0.96
Fat, %	15.21 ^A^ ± 1.49	16.65 ^B^ ± 2.12	5.97 ^A^ ± 0.48	7.27 ^B^ ± 0.48	3.16 ^A^ ± 0.41	3.93 ^B^ ± 0.34	2.27 ^B^ ± 0.6	1.76 ^A^ ± 0.19	3.06 ^A^ ± 0.41	3.84 ^B^ ± 0.25
Energy value, kJ·100 g^−1^	852.20 ± 53.45	896.04 ± 64.53	499.53 ^a^ ± 24.57	555.69 ^b^ ± 56.23	421.02 ^A^ ± 39.98	498.79 ^B^ ± 44.66	464.93 ± 33.67	466.01 ± 35.67	410.72 ± 44.10	409.09 ± 16.19

C—control group; E—experimental group; WHC—water holding capacity; A,B—significant differences in the columns for offal, marked with different capital letters, differ at *p* ≤ 0.01; a,b—significant differences in the columns for offal, marked with different small letters, differ at *p* ≤ 0.05.

**Table 6:** Fatty acids content of the offal.

Traits	Offal Type									
	Tongue		Heart		Lungs		Liver		Kidneys	
	C Mean ± SD	E Mean ± SD	C Mean ± SD	E Mean ± SD	C Mean ± SD	E Mean ± SD	C Mean ± SD	E Mean ± SD	C Mean ± SD	E Mean ± SD
C10:0, %	0.16 ^B^ ± 0.01	0.09 ^A^ ± 0.01	0.12 ^A^ ± 0.01	0.09 ^B^ ± 0.01	0.06 ^A^ ± 0.01	0.09 ^B^ ± 0.01	0.12 ^B^ ± 0.01	0.00 ^A^ ± 0.00	0.05 ^A^ ± 0.01	0.09 ^B^ ± 0.01
C12:0, %	0.09 ± 0.01	0.09 ± 0.02	0.11 ± 0.01	0.11 ± 0.01	0.12 ± 0.01	0.11 ± 0.01	0.17 ^B^ ± 0.06	0.09 ^A^ ± 0.00	0.10 ^A^ ± 0.01	0.14 ^B^ ± 0.02
C14:0, %	1.32 ± 0.17	1.34 ± 0.08	1.13 ^a^ ± 0.02	1.24 ^b^ ± 0.05	2.68 ^B^ ± 0.06	1.85 ^A^ ± 0.17	1.28 ^B^ ± 0.07	0.65 ^A^ ± 0.03	0.91 ^A^ ± 0.02	1.63 ^B^ ± 0.31
C15:0, %	0.04 ^a^ ± 0.01	0.06 ^b^ ± 0.01	0.06 ^A^ ± 0.00	0.09 ^B^ ± 0.02	0.23 ^A^ ± 0.02	0.13 ^B^ ± 0.01	0.18 ^B^ ± 0.03	0.13 ^A^ ± 0.02	0.12 ^B^ ± 0.01	0.09 ^A^ ± 0.02
C16:0, %	25.51 ± 1.83	25.08 ± 0.64	24.20 ^a^ ± 1.15	25.15 ^b^ ± 1.00	35.07 ^B^ ± 0.67	30.48 ^A^ ± 1.30	21.00 ^B^ ± 0.36	19.41 ^A^ ± 0.65	25.14 ^A^ ± 0.70	27.78 ^B^ ± 1.63
C16:1, %	4.08 ^a^ ± 0.35	4.47 ^b^ ± 0.32	2.35 ^a^ ± 0.09	2.55 ^b^ ± 0.07	3.14 ^B^ ± 0.12	2.90 ^A^ ± 0.19	2.19 ^B^ ± 0.21	1.77 ^A^ ± 0.20	1.56 ^A^ ± 0.03	2.02 ^B^ ± 0.11
C17:0, %	0.29 ^B^ ± 0.02	0.25 ^A^ ± 0.03	0.32 ± 0.02	0.32 ± 0.01	0.46 ^B^ ± 0.07	0.33 ^A^ ± 0.02	0.94 ^A^ ± 0.14	1.16 ^B^ ± 0.32	0.62 ^B^ ± 0.02	0.32 ^A^ ± 0.05
C17:1, %	0.37 ± 0.04	0.35 ± 0.05	0.23 ^A^ ± 0.02	0.30 ^B^ ± 0.02	0.28 ^b^ ± 0.01	0.25 ^a^ ± 0.03	0.36 ^A^ ± 0.01	0.42 ^B^ ± 0.05	0.22 ^A^ ± 0.01	0.25 ^B^ ± 0.05
C18:0, %	11.40 ^B^ ± 0.62	9.74 ^A^ ± 0.27	16.72 ^B^ ± 0.98	15.12 ^A^ ± 1.5	15.45 ^B^ ± 0.64	14.74 ^A^ ± 0.69	25.39 ^A^ ± 1.94	26.58 ^B^ ± 2.09	16.99 ^a^ ± 0.40	17.62 ^b^ ± 1.83
C18:1n9c + C18:1n9t, %	50.44 ^a^ ± 1.54	51.49 ^b^ ± 0.78	43.77 ^a^ ± 1.22	45.84 ^b^ ± 1.19	30.46 ^A^ ± 1.49	38.66 ^B^ ± 1.93	25.4 0± 0.52	25.47 ± 1.56	22.17 ^A^ ± 0.48	37.84 ^B^ ± 2.27
C18:2n6c + C18:2n6t, %	3.59 ^A^ ± 0.32	4.38 ^B^ ± 0.49	6.64 ± 0.75	6.12 ± 0.35	4.81 ± 0.33	4.82 ± 0.41	10.00 ^A^ ± 0.58	11.96 ^B^ ± 1.56	13.78 ^B^ ± 0.28	6.40 ^A^ ± 0.75
C18:3n6, %	0.16 ^a^ ± 0.04	0.14 ^b^ ± 0.02	0.08 ^a^ ± 0.02	0.10 ^b^ ± 0.01	0.07 ± 0.01	0.06 ± 0.01	0.07 ^A^ ± 0.01	0.11 ^B^ ± 0.03	0.07 ^B^ ± 0.01	0.00 ^A^ ± 0.00
C18:3n3, %	0.25 ^A^ ± 0.03	0.32 ^B^ ± 0.04	0.27 ^A^ ± 0.02	0.34 ^B^ ± 0.05	0.21 ± 0.05	0.21 ± 0.03	0.28 ^A^ ± 0.06	0.38 ^B^ ± 0.10	0.23 ^A^ ± 0.01	0.47 ^B^ ± 0.06
C20:0, %	0.13 ^a^ ± 0.01	0.15 ^b^ ± 0.03	0.19 ^b^ ± 0.02	0.17 ^a^ ± 0.01	0.16 ± 0.00	0.17 ± 0.03	0.14 ^b^ ± 0.01	0.12 ^a^ ± 0.02	0.29 ^B^ ± 0.01	0.08 ^A^ ± 0.01
C20:1, %	1.65 ^B^ ± 0.51	1.59 ^A^ ± 0.12	1.19 ^A^ ± 0.06	1.27 ^B^ ± 0.03	0.53 ^A^ ± 0.02	0.82 ^B^ ± 0.06	0.30 ^a^ ± 0.01	0.47 ^b^ ± 0.04	0.39 ^A^ ± 0.02	0.66 ^B^ ± 0.06
C20:3n6, %	0.51 ^B^ ± 0.06	0.43 ^A^ ± 0.06	2.54 ^B^ ± 0.32	1.03 ^A^ ± 0.09	5.68 ^B^ ± 0.81	3.76 ^A^ ± 0.48	11.11 ^A^ ± 1.14	10.10 ^B^ ± 1.37	12.78 ^B^ ± 0.32	3.34 ^A^ ± 0.44
C20:4n6, %	ND	ND	0.08 ^b^ ± 0.01	0.03 ^a^ ± 0.01	0.03 ^a^ ± 0.01	0.05 ^b^ ± 0.02	0.38 ± 0.01	0.35 ± 0.09	0.47 ^B^ ± 0.01	0.06 ^A^ ± 0.04
C20:5n3, %	ND	ND	ND	ND	0.10 ^b^ ± 0.01	0.08 ^a^ ± 0.03	0.12 ^b^ ± 0.04	0.01 ^a^ ± 0.01	ND	ND
C22:2n6, %	ND	ND	ND	ND	0.01 ^a^ ± 0.01	0.38 ^b^ ± 0.03	0.01 ^a^ ± 0.01	0.05 ^b^ ± 0.01	ND	ND
C24:0, %	ND	ND	ND	ND	0.43 ^b^ ± 0.04	0.12 ^a^ ± 0.04	0.29 ^A^ ± 0.01	0.36 ^B^ ± 0.10	2.81 ^B^ ± 0.3	1.18 ^A^ ± 0.19

C—control group; E—experimental group; A,B—significant differences in the columns for offal, marked with different capital letters, differ at *p* ≤ 0.01; a,b—significant differences in the columns for offal, marked with different small letters, differ at *p* ≤ 0.05; ND—not detectable.

**Table 7:** Percentage of different groups of fatty acids, their ratios and dietary indexes of the offal.

Traits	Offal Type									
	Tongue		Heart		Lungs		Liver		Kidneys	
	C Mean ± SD	E Mean ± SD	C Mean ± SD	E Mean ± SD	C Mean ± SD	E Mean ± SD	C Mean ± SD	E Mean ± SD	C Mean ± SD	E Mean ± SD
SFA, %	38.95 ^b^ ± 3.58	36.80 ^a^ ± 0.69	42.80 ± 1.01	42.33 ± 1.02	54.71 ^B^ ± 2.87	48.01 ^A^ ± 1.92	49.62 ± 2.21	49.72 ± 1.42	47.11 ± 0.96	47.73 ± 2.05
MUFA, %	56.54 ^a^ ± 1.42	57.92 ^b^ ± 0.50	47.54 ^a^ ± 1.21	50.05 ^b^ ± 1.22	34.40 ^A^ ± 1.42	43.01 ^B^ ± 1.99	28.42 ± 0.66	28.32 ± 1.64	25.55 ^A^ ± 0.46	42.01 ^B^ ± 2.47
PUFA, %	4.51 ^A^ ± 0.38	5.28 ^B^ ± 0.50	9.60 ^B^ ± 0.88	7.62 ^A^ ± 0.44	10.89 ^B^ ± 1.08	8.98 ^A^ ± 0.91	21.96 ^a^ ± 1.71	22.96 ^b^ ± 1.07	27.34 ^B^ ± 1.55	10.26 ^A^ ± 1.02
OMEGA 3, %	0.25 ^A^ ± 0.03	0.32 ^B^ ± 0.04	0.27 ^A^ ± 0.02	0.34 ^B^ ± 0.05	0.30 ± 0.04	0.29 ± 0.06	0.41 ± 0.02	0.38 ± 0.03	0.23 ^A^ ± 0.02	0.47 ^B^ ± 0.09
OMEGA 6, %	4.26 ^A^ ± 0.36	4.96 ^B^ ± 0.48	9.34 ^B^ ± 0.88	7.28 ^A^ ± 0.41	10.580 ^B^ ± 1.11	8.69 ^A^ ± 0.86	21.55 ^a^ ± 1.70	22.57 ^b^ ± 1.02	27.11 ^B^ ± 0.54	9.79 ^A^ ± 1.10
UFA, %	61.05 ^a^ ± 3.06	63.2 ^b^ ± 6.9	57.20 ± 2.00	57.67 ± 1.02	45.29 ^A^ ± 2.54	51.99 ^B^ ± 1.92	50.38 ± 2.21	51.28 ± 1.42	52.89 ± 2.12	52.27 ± 2.05
UFA/SFA	1.57 ^a^ ± 0.11	1.72 ^b^ ± 0.05	1.33 ± 0.05	1.36 ± 0.06	0.83 ^A^ ± 0.03	1.09 ^B^ ± 0.08	1.02 ± 0.09	1.05 ± 0.06	1.12 ± 0.04	1.10 ± 0.09
MUFA/SFA	1.46 ^a^ ± 0.10	1.57 ^b^ ± 0.05	1.11 ^a^ ± 0.05	1.18 ^b^ ± 0.06	0.63 ^A^ ± 0.03	0.90 ^B^ ± 0.07	0.57 ± 0.04	0.58 ± 0.05	0.54 ^A^ ± 0.02	0.88 ^B^ ± 0.09
PUFA/SFA	0.12 ^a^ ± 0.01	0.14 ^b^ ± 0.01	0.22 ^b^ ± 0.02	0.18 ^a^ ± 0.01	0.20 ± 0.02	0.19 ± 0.02	0.44 ± 0.03	0.47 ± 0.03	0.58 ^B^ ± 0.02	0.22 ^A^ ± 0.02
DFA, %	72.45 ± 1.88	72.94 ± 1.88	73.87 ± 2.12	72.79 ± 1.99	60.74 ^A^ ± 3.20	66.73 ^B^ ± 2.44	75.77 ^A^ ± 4.90	77.85 ^B^ ± 1.69	69.88 ± 3.68	69.89 ± 1.75
OFA, %	27.55 ± 1.88	27.06 ± 1.69	26.13 ± 2.12	27.01 ± 1.99	39.26 ^B^ ± 3.20	33.2 ^A^ ± 2.44	24.23 ^B^ ± 4.90	22.15 ^A^ ± 1.69	30.12 ± 3.68	30.11 ± 1.75
AI	0.51 ± 0.05	0.48 ± 0.02	0.50 ± 0.03	0.52 ± 0.03	1.01 ^B^ ± 0.03	0.73 ^A^ ± 0.06	0.52 ^B^ ± 0.03	0.43 ^A^ ± 0.02	0.55 ^A^ ± 0.02	0.66 ^B^ ± 0.02
TI	1.27 ^A^ ± 0.09	1.14 ^B^ ± 0.03	1.55 ^B^ ± 0.06	1.45 ^A^ ± 0.06	2.50 ^B^ ± 0.12	1.89 ^A^ ± 0.16	1.98 ± 0.19	1.96 ± 0.18	2.18 ^B^ ± 0.11	1.75 ^A^ ± 0.22

C—control group; E—experimental group; SFA—saturated fatty acids; UFA—unsaturated fatty acids; MUFA—monounsaturated fatty acids, PUFA—polyunsaturated fatty acids; OMEGA 3—omega-3 polyunsaturated fatty acid; OMEGA 6—omega-6 polyunsaturated fatty acids; DFA—neutral and hypocholesterolemic fatty acids; OFA—hypercholesterolemic fatty acids; AI—atherogenic index; Ti—thrombogenic index; A,B—significant differences in the columns for offal, marked with different capital letters, differ at *p* ≤ 0.01; a,b—significant differences in the columns for offal, marked with different small letters, differ at *p* ≤ 0.05.

**Table 8:** Mineral content (mg·kg^−1^) of the offal.

Traits	Offal Type									
	Tongue		Heart		Lungs		Liver		Kidneys	
	C Mean ± SD	E Mean ± SD	C Mean ± SD	E Mean ± SD	C Mean ± SD	E Mean ± SD	C Mean ± SD	E Mean ± SD	C Mean ± SD	E Mean ± SD
Na	789.17 ^a^ ± 38.75	905.60 ^b^ ± 128.49	1298.76 ± 77.34	1385.10 ± 99.87	1065.33 ^b^ ± 54.66	916.99 ^a^ ± 113.88	787.66 ± 45.45	755.56 ± 59.41	872.37 ± 39.22	810.76 ± 97.25
K	2477.73 ± 112.66	2564.32 ± 129.41	2772.61 ^B^ ± 156.17	2244.16 ^A^ ± 54.06	2556.37 ^B^ ± 129.66	2078.66 ^A^ ± 265.77	2856.88 ± 145.54	2699.40 ± 389.91	2934.70 ^a^ ± 133.39	3348.65 ^b^ ± 397.71
Mg	186.47 ± 19.13	174.71 ± 11.94	167.03 ± 13.22	189.22 ± 9.79	138.31 ± 4.94	114.23 ± 35.40	188.67 ^A^ ± 12.58	246.27 ^B^ ± 25.61	213.55 ± 8.80	225.24 ± 10.10
Ca	132.67 ± 6.63	139.70 ± 9.7	137.05 ^A^ ± 6.9	201.97 ^B^ ± 3.94	84.37 ^A^ ± 2.14	152.93 ^B^ ± 19.28	74.28 ^A^ ± 12.44	114.94 ^B^ ± 21.22	94.55 ± 13.13	101.69 ± 9.77
Fe	15.54 ^A^ ± 0.57	23.32 ^B^ ± 4.81	36.63 ^A^ ± 0.97	65.62 ^B^ ± 2.82	48.93 ^A^ ± 19.97	87.79 ^B^ ± 3.11	177.65 ^A^ ± 10.81	225.49 ^B^ ± 19.72	43.30 ± 3.38	41.87 ± 5.87
Zn	18.77 ± 0.35	19.95 ± 1.66	26.67 ^B^ ± 0.87	20.13 ^A^ ± 0.94	17.68 ^B^ ± 1.10	13.01 ^A^ ± 3.24	50.14 ± 4.31	52.32 ± 15.66	17.01 ± 1.43	18.06 ± 1.54
Cu	2.14 ± 0.20	2.24 ± 0.36	6.16 ^B^ ± 0.24	3.47 ^A^ ± 0.08	1.19 ± 0.03	1.09 ± 0.36	8.88 ± 0.40	10.60 ± 4.99	4.08 ± 0.20	3.93 ± 0.19
Mn	0.54 ± 0.01	0.44 ± 0.06	1.79 ^A^ ± 0.12	1.23 ^B^ ± 0.05	0.17 ^A^ ± 0.01	0.36 ^B^ ± 0.05	3.01 ± 0.14	2.79 ± 0.45	0.45 ± 0.01	0.41 ± 0.09

C—control group; E—experimental group; K-potassium; Na-sodium; Mg-magnesium; Ca—calcium; Zn-zinc, Fe—irons, Mn—manganese; Cu—copper; A,B—significant differences in the columns for offal, marked with different capital letters, differ at *p* ≤ 0.01; a,b—significant differences in the columns for offal, marked with different small letters, differ at *p* ≤ 0.05.

## Data Availability

All data are available from the authors’ database.

## Appendix A. Statement on the Lack of Permission from the Local Ethics Committee

According to Polish legislation—Act of 15 January on the protection of animals used for scientific purposes [24] and the Directive 2010/63/EU of The European Parliament and of The Council of 22 September 2010 on the protection of animals used for scientific purposes [25], research described in the manuscript entitled: “*Effect of Milk Thistle (Silybum Marianum) Supplementation on Pork Offal Quality*” was not a procedure and therefore did not need the approval of the Local Ethics Committee.

There was no legal basis for requesting approval from the Local Ethics Committee to conduct the research because in accordance with Article 2, Paragraph 1, Point 6 [24] and Article 2, Paragraph 4, Point f [25] during the research no activities were performed that could cause pain, suffering, stress or permanent damage to the body equal to or more than a needle stick injury. The animals were slaughtered in accordance with routine procedures of the slaughterhouse.

Moreover, in accordance with Article 1, Paragraph 1, Point 4 [24], the Act does not apply to animal husbandry and breeding conducted in accordance with the provisions on animal protection. The animals used in the experiment came from a farm that complied with all the welfare requirements and fulfilled the nutritional standards [22]. None of the activities performed changed the production cycle.

Furthermore, according to the law in force in Europe and Poland [25,26], Milk Thistle is a feed additive approved for use in feeding pigs. Therefore, the feed used satisfied all the nutritional needs of the animals and did not cause a clinical anomaly or pain, so according to Annex 1 [23] the applied nutrition is not a procedure requiring the approval of the Commission.

The safety of using milk thistle in pig nutrition is confirmed by other experiments [7].

Studies conducted in animals have shown that silymarin is not toxic, however, in doses above 1500 mg per day, it has a laxative effect, resulting from increased production and flow of bile [65].The applied dose of milk thistle, i.e., 7 g, with the silymarin complex content of 2.93% and the daily feed consumption—2.87 kg, gives the consumption of silymarin at the level of approx. 770 mg/day, which is almost two times lower than the dose showing negative effects. Therefore, another argument in favor of the lack of the need for approval of the Ethics Committee for the research is that the purpose of our study was not to determine the permissible maximum dose of milk thistle in pig nutrition (exceeding this dose could cause pain and anomalies), but to determine the effect of a non-toxic amount of milk thistle supplement in pig nutrition on the consumer quality of offal.
